# Variation in CFTR-dependent ‘β-sweating’ among healthy adults

**DOI:** 10.1371/journal.pone.0265432

**Published:** 2022-03-21

**Authors:** Lesje DeRose, Jeeyeon Kim, Miesha Farahmand, Meagan Y. Shinbashi, Nam Soo Joo, Jeffrey J. Wine

**Affiliations:** 1 Cystic Fibrosis Research Laboratory, Stanford University, Stanford, California, United States of America; 2 Department of Pediatrics, Stanford University School of Medicine, Stanford, California, United States of America; 3 Department of Psychology, Stanford University, Stanford, California, United States of America; Heidelberg University, GERMANY

## Abstract

The genetic disease cystic fibrosis (CF) results when mutations in the gene for the anion channel CFTR reduce CFTR’s activity below a critical level. CFTR activity = N·P_O_·γ (number of channels x open probability x channel conductance). Small molecules are now available that partially restore CFTR function with dramatic improvements in health of CF subjects. Continued evaluation of these and other compounds in development will be aided by accurate assessments of CFTR function. However, measuring CFTR activity *in vivo* is challenging and estimates vary widely. The most accurate known measure of CFTR activity *in vivo* is the ‘β/M’ ratio of sweat rates, which is produced by stimulation with a β-adrenergic agonist cocktail referenced to the same individual’s methacholine-stimulated sweat rate. The most meaningful metric of CFTR activity is to express it as a percent of normal function, so it is critical to establish β/M carefully in a population of healthy control subjects. Here, we analyze β/M from a sample of 50 healthy adults in which sweat rates to cholinergic and β-adrenergic agonists were measured repeatedly (3 times) in multiple, (~50) identified sweat glands from each individual (giving ~20,000 measurements). The results show an approximately 7-fold range, 26–187% of the WT average set to 100%. These provide a benchmark against which other measures of CFTR activity can be compared. Factors contributing to β/M variation in healthy controls are discussed.

## Introduction

**Fig 1:**
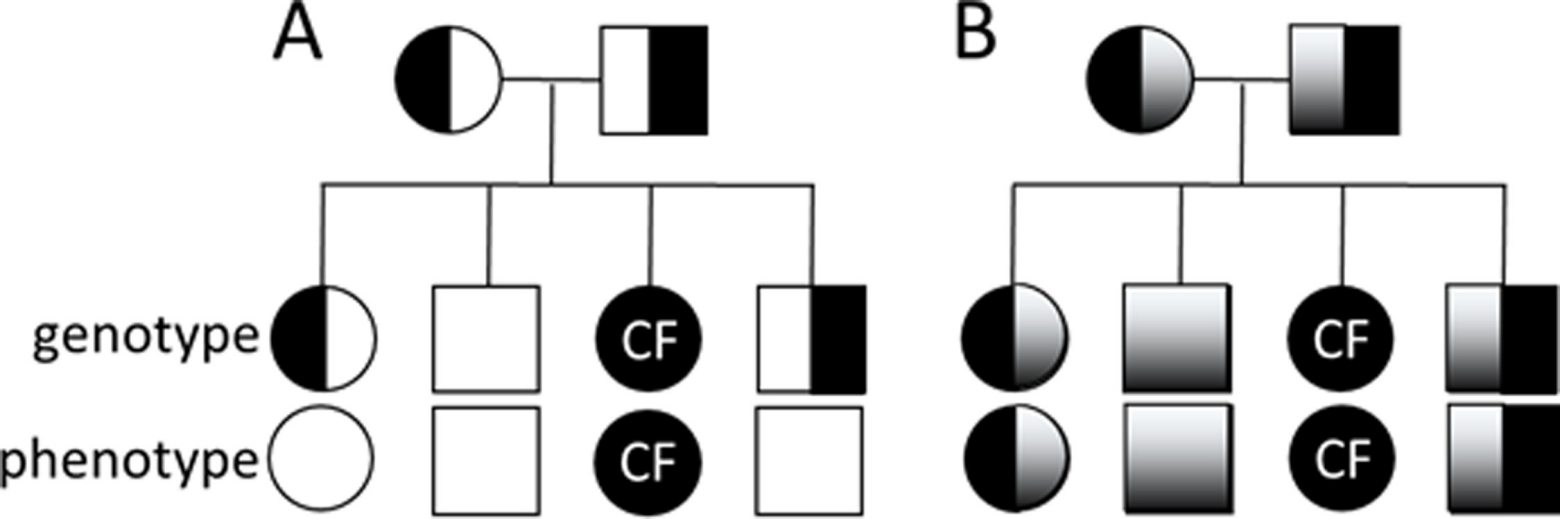
Inheritance probabilities for a monogenetic disease. **A**. Traditional diagram of inheritance probabilities for a monogenetic disease like CF in a family where both parents are carriers. Each child has an equal chance of getting the CF allele, so on average half the children are carriers, ¼ are not carriers, and ¼ have the CF phenotype. This works for phenotypic traits that are not rate-limited by the gene product, so that the carrier phenotype is unaffected, giving on average ¼ affected offspring. **B**. Ranges of phenotypic traits that are rate-limited by the gene that causes disease when both alleles are mutated. Unaffected individual show a range of function and carriers have half of that. For simplicity, a single range of function is shown. White is full function, black is zero function, gradient is intermediate function. CF is a recessive disease in which some traits are rate-limited by the gene product and are therefore ‘semi-dominant’. So far, very few physiological measures are known to be rate-limited by CFTR; these include β-sweat secretion and bicarbonate secretion in the airways.

Autosomal recessive genetic diseases typically occur with loss-of-function mutations in which a single unaffected allele produces enough function to prevent disease. This concept is illustrated by diagrams like **Fig 1A**, in which offspring from two unaffected carriers have a ½ chance of being carriers, ¼ chance of having the disease, and ¼ chance of being free of the disease-causing allele. It was long understood that these diagrams do not capture the full range of phenotypic variation. In the molecular age, ‘simple, monogenetic’ diseases are better termed as ‘simpler, mainly monogenetic’ diseases, in which modifier genes, environmental factors and variable activity of the normal gene contribute to the distribution of phenotypes encountered [1]. For phenotypic traits that are rate-limited by the relevant gene product, the situation is better described by **Fig 1B**.

Cystic fibrosis (CF) is a recessive genetic disease for which a deep and nuanced understanding of phenotypic variation has been achieved. CF is caused by reduced activity in CFTR, a Cl^-^ and HCO_3_^-^ channel mainly found in epithelia. CFTR activity = the number of channels in the membrane, times their open probability, times their conductance (N·P_O_·γ). Mutations in the CFTR gene can affect any of these. Disease presentation among affected individuals varies widely. ‘Unaffected’ carriers are also at increased risk for some conditions associated with CF. In a recent study using a massive database, 19,802 CF carriers were studied. Each was matched with five controls, and the prevalence of 59 CF related conditions was evaluated in each cohort. All but two of the 59 CF-related conditions were found to have significantly increased risk (p < 0.05) in the CF carrier cohort [2]. This is consistent with these traits being rate-limited by CFTR activity.

Factors that contribute to variation in the CF phenotype vary by organ system and by what is measured. For example, variation in lung function, which is the most limiting condition for most people with CF, is determined mainly by chronic lung infections; if these are prevented health and longevity increase dramatically [3, 4]. However, as expected for a monogenetic disease, variations in CFTR activity dominate variation for most systems and measures, although to a lesser extent than might be thought. For example, in a study of 2,639 sweat chloride measurements from 1,761 twins/siblings, CFTR gene mutations accounted for 56.1% of variation in the sample, with the rest of the variation coming from various sources related to testing and environment. For an individual with CF, 58% of the variation occurred with testing over time, and 42% from residual/random factors [5].

In addition to disease causing mutations, many polymorphisms exist in the large CFTR gene, some of which affect CFTR activity. That will contribute to variation in CFTR-dependent traits in the non-CF, non-CF carrier population, hereafter referred to as healthy control (HC). Although this is well known, the magnitude of the variation relative to other sources of variation is not known. In this report we used the ‘β-sweat’ assay, which is rate-limited by CFTR activity [6–10], to quantify the variation in β/M among a sample of healthy adults, in an attempt to apportion the variation between CFTR activity and other sources of variation. This is important, because as we analyze trials of modulators that directly alter CFTR activity, it is necessary to consider variation in CFTR activity of healthy controls as one factor that can influence outcomes. In an attempt to distinguish subject and experimental variability, we used multiple measures from multiple identified glands in each subject. The results show that average β/M values ranged from less than half to more than twice the mean value in this sample, with a coefficient of variation of 36%. However, similar variation was observed across individual glands within subjects. Thus, using this assay and a sample size of 50 healthy controls, and without independent measures of CFTR genetic variation, we were unable to apportion variation of β/M between genetic variation of CFTR and various sources of random variation. Nevertheless, we establish the parameters of variation using this assay, and acknowledge that differences in CFTR activity are only one determinant of the variation. Further investigations of the range of CFTR activity in healthy populations are warranted. Implications for health, especially among carriers, is discussed.

## Materials and methods

### Subjects

The study was approved by the Institutional Review Board of Stanford University (IRB #10858). After written informed consent, 68 healthy adult subjects were recruited. Four carriers (3 female), parents or genotyped siblings of CF subjects, were also tested but their data was excluded. Data from 13 subjects, all done sequentially by the same operator, had a series of issues that led to significantly higher deviations in the data compared with all prior subjects. Data from these subjects was excluded. The remaining 50 subjects (33 females, 17 males) were used to determine normal variation in cholinergic and β-adrenergic sweating. Two subjects were tested only once, one subject was tested twice, and the remaining 47 subjects were each tested 3 times. For each subject we measured 68 ± 17 single gland responses to methacholine and 64 ± 19 single gland responses to a β-adrenergic cocktail on each test.

Most subjects were not genotyped so it was necessary to do an ad hoc estimate of the number of potential carriers in the sample. The incidence of carriers in the U.S. is estimated to be 1 in 31 or 0.032, giving probabilities of carriers in a sample of 50 healthy controls as follows: 0: 19.5%, 1: 32.4%, 2: 26.4%, 3: 14.1% and 4: 5.5% (cumulative probability 97.9%, binomial distribution). The distributions of M-sweat, β-sweat, and the β/M ratio of the sample did not depart from normal distributions (Kolmogorov-Smirnov Test). Prior work on smaller samples (n = 19–20) indicated that the carrier mean was one half the control mean with overlap between the top half of carriers and bottom half of control population [6]. To estimate of the consequences of having 1–4 carriers in our sample we converted up to 4 control values below the control mean to 2X values on the assumption that they were actually carriers. Doubling the four lowest values increased the β/M mean ± SD from 0.193 ± 0.070 to 0.199 ± 0.063 (3%). The distribution still did not differ from a normal, but skewness and kurtosis increased 2–3 times. On the more likely assumption that any carriers would be randomly distributed within the lower half of the distribution, four values selected randomly from the lower 25 subjects were doubled in 3 simulations. These increased the mean from 0.193 to 0.203–0.206 (5–6%) with modest effects on skewness and kurtosis. (The study [6] that determined carriers had half normal β sweat rates could also have included carriers control population (n = 19) as follows: 0 carriers: 54%, 1 carrier: 34%, 2 carriers: 10%, 3 carriers: 2%; that study was completed before the CFTR gene had been found.

### Reagents

Methacholine chloride, (Methapharm, Ontario, Canada), isoproterenol HCl, aminophylline, lactated Ringer’s (Hospira, Lake Forest, IL) and atropine sulfate, (American Reagent) were obtained from Stanford University Hospital Pharmacy. Heavy mineral oil was from EMD Chemicals, Gibbstown, NJ. Erioglaucine disodium salt (CAS No. 3844-45-9) was from Sigma.

### Drug delivery and imaging of sweating

For details see ref [11]. In brief, an imaging site on the volar surface of the forearm was chosen, swabbed with alcohol, and then injected intradermally with 0.1 ml of a 1 μM solution of methacholine (MCh) in lactated Ringers using a 30 gauge needle and a 1 ml BD Ultra-Fine syringe. After injection, a 0.3 cm deep reservoir (Sylgard with a hard plastic shell) with internal area of 1.2 cm^2^ was secured over the injection wheal, the skin within the reservoir was dried with compressed gas, and 350 μl of water-saturated mineral oil [12] was added to the reservoir. A ring of light emitting diodes 0.5 cm above the skin surface produced oblique lighting to visualize the unstained methacholine-stimulated sweat bubbles (M-sweat). (Dye was omitted to minimize dye carryover to the cocktail-stimulated (β-sweat) trial.) The reservoir was secured in fixed register with a computer-controlled digital camera equipped with a macro lens. Images were taken at 30 sec intervals. A calibration grid (0.5 mm^2^) was included at the side of the reservoir. The camera imaged an area 7 x 9.5 mm (66.5 mm^2^). The secreted sweat formed expanding spherical bubbles that remain attached to the column of sweat in the openings of the sweat duct and did not wet the oil-covered skin surface. After 10 min (reduced from 15 min used in ref [11]), the sweat and oil are removed, then the reservoir was removed and the area gently blotted with absorbent dressing.

The same site was then re-injected within 2 min with a cocktail of 140 μM atropine, 80 μM isoproterenol and 10 mM aminophylline dissolved in lactated Ringers to make a final injection volume of 0.1 ml. This cocktail stops sweating produced by MCh instantly and elicits a pure β-adrenergic sweat response, as indicated by its total block by propranolol [7]. Two min after cocktail injection, before β-sweating started, the site was rinsed thoroughly with a stream of distilled water and dried with a stream of gas before adding the imaging chamber and the indicator oil 3 min post injection. To help visualize sweat bubbles, particles of a water-soluble dye were dispersed in the oil. When a dye particle touched a sweat bubble, it partitioned into it and stained it uniformly with a bright blue color.

### Stimulation and imaging protocol overview

The assay for CFTR secretory function consisted of two sequential periods of stimulated secretion. The first period (10 min) measured the response to MCh and the second period (30 min) measured the response to cocktail. The increased volumes of individual identified glands were plotted over time in each condition and rates calculated for each gland and for the average. The stimulation paradigm was based on Sato and Sato [7] and the imaging method was adapted from methods developed for airway submucosal glands [12, 13].

### Measurement of sweat secretion

For both M- and β-sweating, images were measured using ImageJ *(*rsbweb.nih.gov/ij/) as described [12, 13]. Sweat bubbles were counted and given identifying numbers. For each identified gland, the circumference of its secreted sweat bubble was measured at a magnification of 250-260X, and was converted to a volume using the formula for a sphere. Average sweat rates for individual glands were determined by calculating the volume secreted per unit time. To reduce the analysis burden we used final sweat volumes to make inter-subject comparisons, with ratios = (30 min β-sweat volume/2) / (10 min M-sweat final volume). The kinetics of β- and cholinergic-stimulated sweat rates were not reported. In the first study of this method [6] rates were recorded at 5 min intervals for 49 glands in a male subject. That showed that cholinergic rates leveled off at 10 min and the β-rates at a similar time. Thus averaging rates over the whole time period underestimates rates for β-sweating relative to cholinergic, but our interest is in comparing the β/M ratios across glands and subjects, for which average rates are suffice. Also, average rates are preferred for measuring β/M ratios in CF subjects on modulators, where β-sweating is very low and often delayed.

### Measurement of sweat chloride

Sweating was induced with pilocarpine iontophoresis using the Macroduct® sweat collection system (Webster sweat inducer- Model 370, UT/USA). We measured chloride levels in the collected sweat using the QuantiChrome^TM^ Chloride Assay kit (Bioassay Systems, CA/USA) and protocol. A subject’s sweat sample was diluted 20-fold in distilled-deionized water and 5 μL of duplicated diluted samples and 5 μL of diluted chloride standard solutions (from the kit) were transferred into wells of a clear bottom 96-well plate. A calibration curve for standards was prepared by diluting standards using double distilled water (0–35 mg/dL). Then, 200 μL of working reagent (from the kit) was added to each well of the diluted sweat samples and standards. After incubating and shaking for 5 min at room temperature, optical density was measured at 610 nm with a SPECTRA max microplate reader (Molecular Devices, CA/USA) and the chloride concentration of each subject’s sweat sample was determined. The measurements were performed in duplicate.

### Statistics

Correlations were assessed with Pearson’s r and differences between means with Student’s t-test. The p values < 0.05 were considered significant.

## Results

### Individual glands: Identification and repeated measures

**Fig 2:**
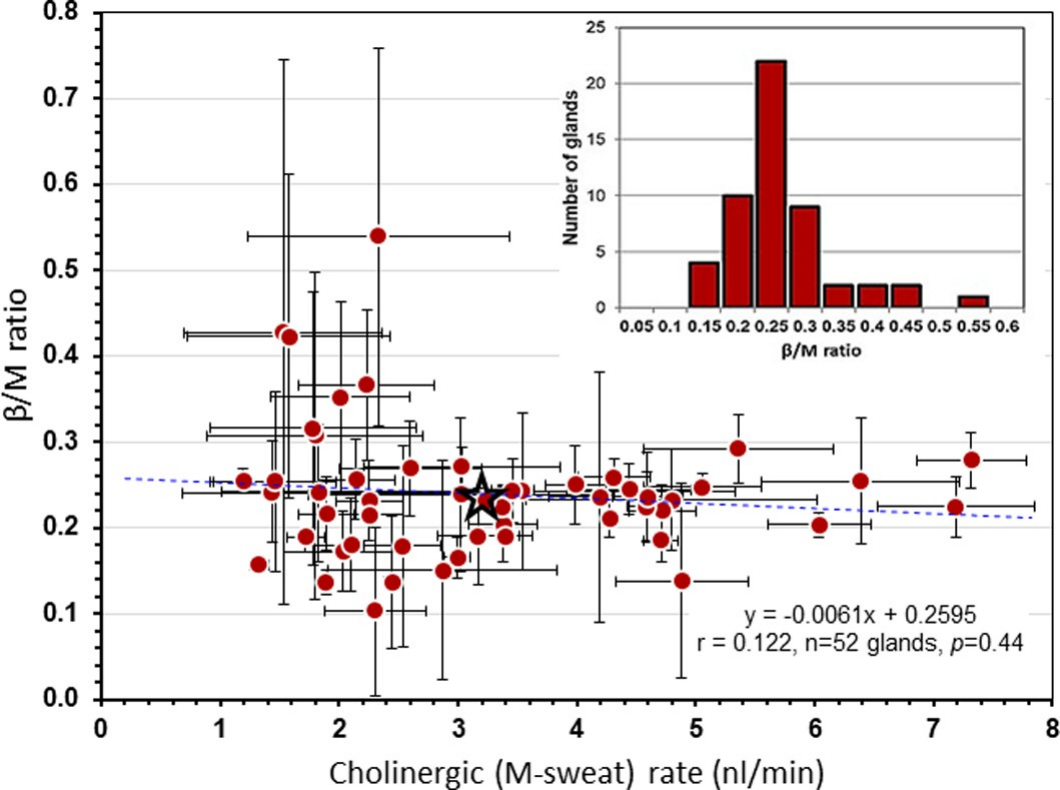
Within-subject sweat rates. Within-subject variation of β-adrenergic/cholinergic sweat rate ratios from each of 52 identified glands in a single subject, S-38, plotted against the cholinergic rate. For each gland, the average across all 3 tests was determined for M and C sweat rates, which was used to compute the Cav/Mav ratio for that gland. The ratio was plotted against the Mav for that gland to give the distribution shown for all 52 glands; means and SDs are plotted. Star shows the grand mean, obtained by summing the Mav from each of the 52 glands dividing by 52 to give (for this subject) 3.2 nl/min/gland. Similarly, the Cav/Mav ratio for each of the 52 glands were summed and divided by 52 for to give (for this subject), Cav/Mav = 0.23. The grand means were plotted for each of 50 subjects in Fig 3. Inset shows the distribution of βav/Mav ratios.

For each subject tested, sweat rates to both cholinergic and β-adrenergic stimulation were optically quantified from a set of identified glands and averaged for each gland and for each subject across 3 tests spaced at weekly intervals. Glands were identified by position relative to a landmark and to one another. Data for 52 glands from one subject are plotted in **Fig 2**. Each point on the graph is the average of three measures for one identified gland; tests for this subject were spaced about one month apart. The glands showed a large range of average responses to methacholine (x-axis) as expected based on different gland sizes, with larger glands having greater secretory capacity [14]. The y-axis plots the distribution of the ratio of CFTR-dependent, β-sweat responses (‘β-sweat’) and CFTR-independent cholinergic responses to methacholine (‘M-sweat’): β/M. The average ratio across all glands mainly reflects CFTR activity adjusted for gland size, because CFTR activity is rate-limiting for β-sweating and β/M drops to zero in almost all individuals with pancreatic insufficient CF (whose cholinergic responses are intact). The star and gray arrows show the overall distribution for the β/M ratio for this subject. The standard deviations generally increase as the averaged responses for each single gland depart from the overall average. Test-by-test variation is mainly experimental error; see Discussion. The slope of β/M over M-sweat rate does not differ from zero, showing that the ratiometric measure compensates well for differences in gland size/secretory capacity.

### Distribution of β/M vs. M-sweat rate across 50 subjects

**Fig 3:**
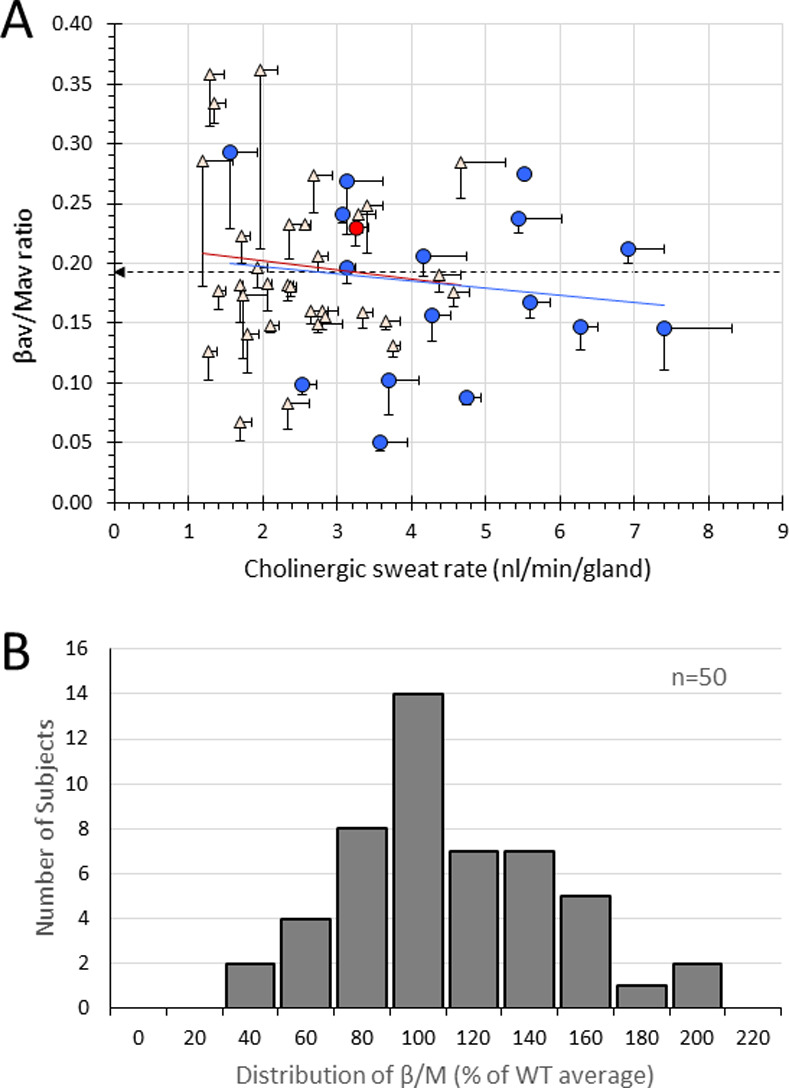
Consistent differences in β-sweating across subjects. **A**. Distribution of average β-sweat rate to the average cholinergic sweat rate for 50 subjects. SEMs are shown in one direction for clarity. Except as noted, each point represents averages from 3 tests per subject and corresponds to the star in **Fig 2**. For each of the three tests an average of 60 ± 19 identified glands were measured. Regression did not differ significantly from zero (r = 0.15, n = 50, p = 0.30) or for sexes considered separately (shown as red and blue lines). Dark dashed arrow indicates the overall β/M average for all 50 subjects; lighter dashed arrows are ± one SD. Pink triangles are female subjects and blue circles are males. Red circle is mean data for subject 38 and corresponds to the star in **Fig 2**. One subject has only two tests and two subjects had only a single test (see **Table 1**). **B**. Each point was normalized to the mean set to 100%, and the distribution plotted.

The grand average across all tests for all glands for a given subject (the star in Fig 2) was plotted for each of 50 subjects, with unidirectional SEM values shown (**Fig 3A)**. Each point represents the average of all single gland measurements for one subject, as in the example shown in **Fig 2**, with the distribution now reflecting consistent differences in average responses across the subjects, based on three tests at weekly intervals for 47 of the 50 subjects. Over a broad range of cholinergic sweat rates, the average β/M ratio showed no significant trend. For example, the average M-sweat rate for females was significantly less than for males, but their β/M ratios did not differ. In nl/min/gland, females: 2.5 ± 0.96, n = 33, vs males, 4.37 ± 1.62, n = 17, p < 0.001. As determined previously [6], the β /M ratio eliminated the sex difference: β/M ratio, female: 0.199 + 0.07 vs. male 0.18 + 0.07, p = 0.47, n.s.

**Table 1:** Subject characteristics and summary data for subjects tested during assay development.

Subj #	Sex	glands MCh (n)	glands β Cktl (n)	Mav	SEM	βav	SEM	βav /Mav	SEM	% WT mean
2	M	49	46	3.13	0.12	0.614	0.019	0.197	0.013	101.8
3	M	63	59	2.54	0.19	0.248	0.007	0.099	0.008	51.0
4	F	60	55	1.40	0.10	0.246	0.005	0.177	0.016	91.7
5	F	64	60	1.35	0.15	0.457	0.072	0.334	0.018	172.8
7	F	77	74	2.34	0.30	0.187	0.041	0.083	0.022	43.1
8**	F	86	88	2.57	0.07	0.60	0.017	0.233	0.001	120.4
9	M	71	85	5.60	0.27	0.934	0.039	0.168	0.013	86.7
10	F	75	69	2.10	0.12	0.312	0.016	0.148	0.006	76.7
11	F	41	42	4.56	0.23	0.806	0.092	0.174	0.016	90.1
12	F	100	111	2.35	0.21	0.541	0.055	0.233	0.030	120.5
13	F	56	53	2.66	0.22	0.422	0.008	0.161	0.011	83.0
14	F	87	105	3.66	0.19	0.553	0.012	0.152	0.007	78.4
15	F	78	76	1.96	0.24	0.708	0.300	0.362	0.150	187.1
16	F	84	86	3.75	0.11	0.494	0.048	0.132	0.011	68.1
17	F	74	80	1.93	0.13	0.375	0.014	0.197	0.017	101.7
18	F	49	49	4.67	0.59	1.304	0.140	0.284	0.030	146.8
19	F	56	55	2.34	0.08	0.426	0.041	0.182	0.014	94.1
20	M	24	24	7.40	0.92	1.015	0.125	0.146	0.034	75.3
21	M	40	42	3.69	0.42	0.398	0.144	0.102	0.028	52.8
22	M	51	46	4.17	0.58	0.840	0.064	0.206	0.016	106.4
23	M	39	38	3.13	0.49	0.804	0.064	0.268	0.044	138.8
24	F	71	70	1.69	0.17	0.119	0.039	0.067	0.015	34.7
25	F	84	84	3.35	0.14	0.535	0.060	0.159	0.014	82.3
26	F	87	96	2.75	0.32	0.406	0.026	0.150	0.008	77.3
27	F	71	71	1.71	0.13	0.375	0.020	0.223	0.022	115.1
28	F	73	91	1.19	0.40	0.269	0.050	0.286	0.105	147.9
29	F	68	68	4.38	0.29	0.836	0.108	0.190	0.014	98.2
30	F	75	73	3.28	0.24	0.784	0.013	0.241	0.016	124.8
31	F	95	95	2.37	0.07	0.428	0.015	0.181	0.009	93.5
32	M	59	58	5.44	0.59	1.277	0.076	0.237	0.011	122.5
33	M	76	76	4.27	0.27	0.666	0.099	0.156	0.021	80.8
34	F	54	59	2.69	0.24	0.723	0.029	0.274	0.032	141.7
35	F	68	65	2.81	0.20	0.444	0.020	0.160	0.016	82.8
36	M	61	58	3.57	0.38	0.177	0.027	0.050	0.008	26.0
37	F	74	72	2.75	0.14	0.564	0.035	0.206	0.014	106.4
38	M	71	69	3.25	0.17	0.743	0.018	0.230	0.015	118.9
40	M	58	55	6.28	0.24	0.913	0.084	0.147	0.019	75.9
41	F	94	92	2.06	0.05	0.378	0.054	0.183	0.023	94.4
42	M	46	45	1.56	0.36	0.412	0.040	0.293	0.064	151.6
44	F	48	49	1.69	0.04	0.310	0.059	0.182	0.032	94.2
46*	M	40	65	5.53		1.52		0.275		142.4
47	M	50	51	4.75	0.18	0.419	0.039	0.088	0.006	45.4
48	F	57	56	1.27	0.12	0.156	0.019	0.127	0.024	65.5
49	F	51	51	3.40	0.22	0.835	0.119	0.248	0.039	128.3
50	F	54	54	1.73	0.33	0.268	0.028	0.174	0.053	89.8
51	F	55	54	1.29	0.18	0.446	0.009	0.359	0.044	185.3
52	M	42	40	3.06	0.04	0.740	0.031	0.241	0.007	124.7
53*	F	57	57	2.84		0.440		0.155		80.1
54	F	46	57	1.80	0.15	0.249	0.056	0.140	0.031	72.6
55	M	52	50	6.93	0.48	1.457	0.027	0.212	0.012	109.5

* indicates tested once and

** tested twice. The remainder were tested 3 times at weekly intervals or more.

Each individual’s β/M ratio was then divided by the overall β/M ratio mean of 0.1934 and multiplied by 100 to give the distribution plotted in **Fig 3B.** The summary statistics for this distribution are shown in **Table 2**.

**Table 2:** Summary statistics for βav/M and % WT.

STATISTIC	ΒETA/M VALUE	%WT
**MEAN**	0.193	100.00
**STANDARD ERROR**	0.01	5.15
**MEDIAN**	0.18	94.15
**STANDARD DEVIATION**	0.07	36.42
**SAMPLE VARIANCE**	0.005	1326.4
**KURTOSIS**	0.083	0.083
**SKEWNESS**	0.38	0.38
**RANGE**	0.31	161.15
**MINIMUM**	0.05	25.98
**MAXIMUM**	0.36	187.13
**COUNT**	50	50

### Sweat chloride measurements

**Fig 4:**
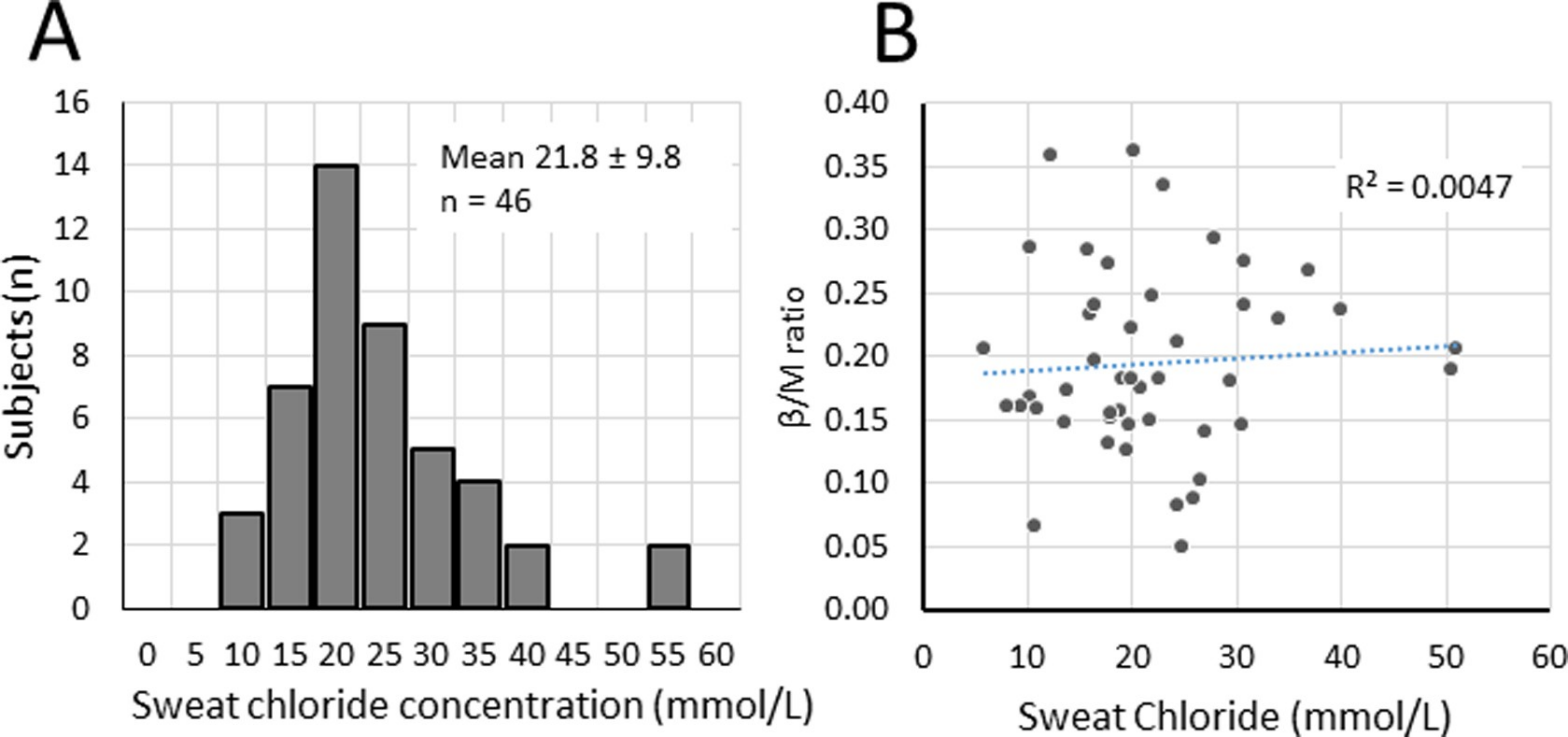
Distribution of sweat chloride values for 46 control subjects. **A**. Distribution of sweat chloride values for 46 control subjects. The distribution does not differ significantly from normal (p = 0.63, Kolmogorov-Smirnov test). **B**. Sweat chloride values were uncorrelated with β/M values (r = 0.07, n = 46, p = 0.65).

Usable sweat chloride values were obtained for 46 of the subjects (**Fig 4A)**. Subjects were tested only once, on one arm. The mean volume collected was 50.07 ± 22.85 μL. Mean sweat chloride was 21.8 ± 9.8 mmol/L, median was 20.0 mmol/L. The cutoff value for a normal reading is < 30 mmol/L and the cutoff for a diagnosis of CF is >60 mmol/L. Two readings were at the high end of the intermediate range, but these subjects had average β/M ratios, and when all measures were regressed against β/M values we found no correlation (**Fig 4B**). Sweat chloride values for females (n = 30) were significantly lower than for males (n = 16): 18.4 ± 8.1 vs. 28.8 ± 9.6 mmol/L, p < 0.01. Sweat chloride values are known to be lower at slower sweat rates [15], but the aggregate sweat volumes did not differ significantly by sex: females = 49.1 ± 22.2 vs. males 53.1 ± 24.7 μl, p = 0.63. However, female glands tend to be smaller and more numerous than male glands (see Discussion), so the aggregate volume obscures *glandular* sweat rates, which are what matters with regard to sweat chloride levels. Average glandular secretion rates to methacholine injection (M-sweat) were significantly lower for females. In nl/min/gland, females: 2.5 ± 0.9 vs males 4.7 ± 1.3, n = 31 and 14 respectively, p < 0.001.

## Discussion

The results show a wide range of CFTR-dependent β-sweating in our control population after normalization to cholinergic sweating in the same glands. These results are consistent with our original results obtained with different methodology [6]. Others have also reported wide variation in β sweating [7–10], but they did not normalize to cholinergic sweating, so variations in cholinergic sweating will be additive with variation in the CFTR-dependent sweat rate. We hypothesized that the residual variation after normalization to cholinergic sweating, i.e. the β/M ratio, would mainly reflect variation in CFTR activity because CFTR is rate limiting for β-sweating. However, because of other sources of variation our results can neither support nor reject the hypothesis.

### Overview of sweat gland secretory cells

The molecular basis of eccrine sweat secretion is imperfectly understood, but a brief review of what is known can help inform attempts to determine sources of variation in the β/M sweat ratio. The sweat coil contains two types of secretory cells: clear and dark cells. Both cell types respond to cholinergic stimulation, but only the clear cells respond to β-adrenergic agonists. The β-sensitive clear cells are the major source of secretion to cholinergic stimulation and the sole source of β-sweat via CFTR. Clear cells have features to support large and sustained anion efflux through CaCC channels in response to cholinergic stimulation including a large and regulated complement of Na^+^, K^+^, 2 Cl^-^ cotransporters, calcium-activated K^+^ channels, abundant mitochondria, intercellular canaliculi and a tubulo-cisternal endoplasmic reticulum. Cholinergic stimulation hyperpolarizes the cells and produces large volume decreases, large drops in K^+^ and Cl^-^ concentrations, and a 4.3 fold increase of Na^+^, consistent with activity of NKCC [16–19].

By contrast, β-stimulation *depolarizes* the clear cells. The electrochemical driving force for anion efflux through CFTR is apparently K^+^ flux through non-regulated (‘leak’) K^+^ channels; there are no cAMP-stimulated K^+^ channels. The maximal β-sweat rate is ~20% of the maximal M-sweat and it declines rapidly after intradermal drug injections while M-sweat remains elevated, so that after 20 min the volume of secreted M-sweat is 11-fold greater than the volume of β-sweat. How Cl^-^ is loaded during β-sweating is not known, indeed, it isn’t even known if β-stimulated sweat is primarily mediated by Cl^-^; HCO_3_^-^ secretion is a possibility. However, if Cl^-^ is the main anion, loading would most likely be via NKCC, present in abundance to serve M-sweating via CaCC. (See refs: [18, 20–23]).

Even less is known about the dark cells, which seem to be the source of most proteins in sweat. They hyperpolarize when stimulated with ACh, but show little or no cell shrinkage; lack a tubulocisternal ER and probably lack CFTR. Because they do not respond to β-agonists they contribute only to M-sweat—perhaps a minor component based on their known features. They are outnumbered 2:1 by clear cells [17].

### Sources of variation in cholinergic sweat rates

**Fig 5:**
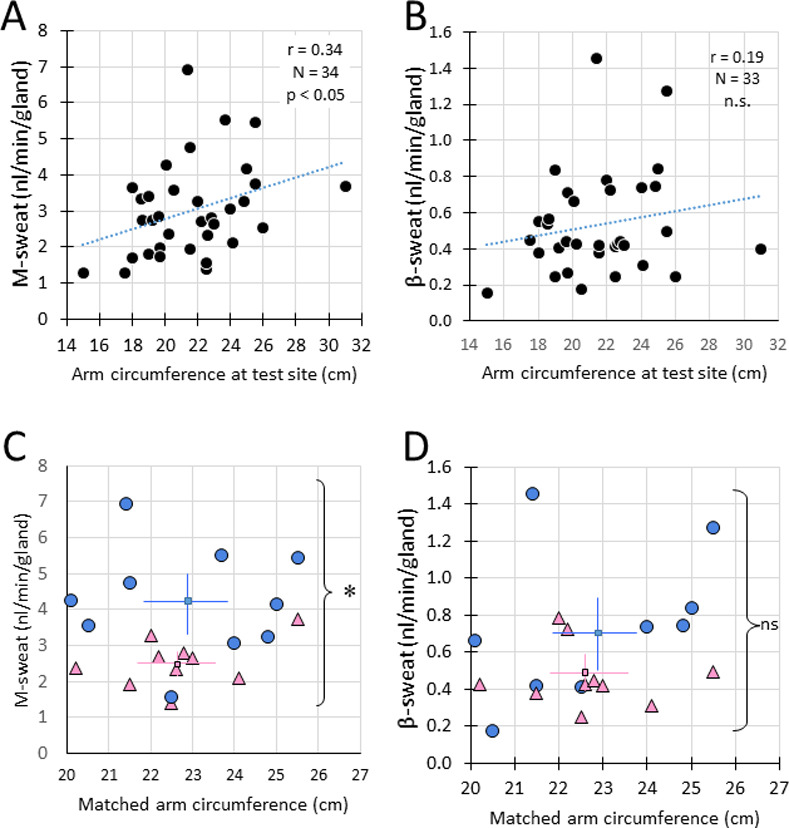
Arm size and sex affect sweat gland M-sweat rates. **A**. Arm circumference at the test site was positively correlated with M-sweat rates of individual glands (r = 0.34, n = 34, p < 0.05). **B**. Arm circumference at the test site was positively correlated with C-sweat rates of individual glands but did not reach significance (r = 0.19, n = 33, n.s). **C.** In a size-matched sample, M-sweat rates of males (blue circles) were significantly greater than females (pink triangles), 2.53 ± 0.67 vs. male: 4.25 ± 1.52, p < 0.01,*indicates p < 0.01. Means and SD indicated. **D**. In a size-matched sample, β-sweat rates of males (blue circles) were not significantly greater than females (pink triangles): female 0.47 ± 0.17 vs male 0.75 ± 0.41 p = 0.08, ns). Means and SD indicated.

Normal sweating for thermal regulation, mediated by cholinergic pathways, was used to calibrate β-sweating, so it is important to consider factors that influence the cholinergic sweat rate. Two factors were apparent in our data: sex (male glands secreting at higher rates on average) and gland size/secretory capacity, which is inversely related to gland density. These two measures are confounded in typical measures of sweat rates that are referenced to an area of skin, such as evaporimetry. Gland density per unit area of skin decreases with body size. This comes about because the number of sweat glands is fixed at birth or shortly thereafter [24]. Therefore, maintaining adequate sweat production per unit area of skin requires increased output per gland (increased gland size). We tested this idea by measuring the arm circumference at the monitored site for 34 subjects and comparing it to the MCh-stimulated glandular sweat rate. Arm circumference has two components, the size of the arm and the position of the fixed monitoring site on the arm which becomes smaller toward the wrist; we did not attempt to dissociate the contributions of total arm size from positional differences. We found a positive correlation between circumference and M-sweat rate (r = 0.34, n = 34, p < 0.05, **Fig 5A**). Cocktail-stimulated sweat rates did not increase significantly with arm circumference, (r = 0.19, n = 33, p = 0.29, **Fig 5B**). We also plotted the number of active glands (MCh-stimulated sweat bubbles) in our unit area vs. arm circumference at the imaging site. The correlation seen between gland number and arm circumference had a negative slope, but it was not significant (r = 0.29, n = 34, p = 0.09). The negative trend may have been blunted because not all glands were measured when the count was significantly higher than 50 glands.

We tried to distinguish between sex and size differences in sweat rates. Male arm circumference was significantly larger in our sample (male: 23.8 ± 3.0 cm, n = 12 vs. female: 20.4 ± 2.5 cm, n = 22, p < 0.01). To see if this could account for the sex difference in M-sweat rates, we selected 20 subjects (10 female) with similar arm circumferences at their test sites so that the mean circumferences for the groups did not differ significantly (female: 22.64 ± 1.43 cm vs. male: 22.9 ± 1.96 cm, p = 0.73). For these size-matched groups, the methacholine-stimulated sweat gland rates still differed significantly by sex: in nl/min/gland female: 2.53 ± 0.67 vs. male: 4.25 ± 1.52, p < 0.01, **Fig 5C**. β sweat rates for females were also lower for these same glands, but not significantly: female: 0.47 ± 0.17 vs male 0.75 ± 0.41 p = 0.08, **Fig 5D**. The sex differences were eliminated by normalizing glandular β sweat rates to cholinergic sweat rates. β/M ratios expressed at percent of the overall sample average were: males, 95% ± 37%, n = 17; females, 103% ± 36%, n = 33, p = 0.47.

### Within-subject variation in β/M

**Fig 2** illustrates the variation within single glands to repeated measures, and across glands for both M-sweat and the β/M ratio. Because all glands share the same genotype, *within-gland* variation (across tests, represented by error bars for each gland shown in **Fig 2**) reflects experimental variability related to dispersal of agonists from the injection site, measurement errors, time-dependent changes within glands or subject, and other sources of random variation. *Within subject*, gland-by-gland variation in mean β/M also can’t result from genetically coded differences at the *CFTR* locus. We know that glands vary considerably in size, which the β/M ratio is intended to control, and CFTR channel number per gland could vary because of gland-to-gland differences in the proportion of clear to dark cells, if dark cells actually make a significant contribution to M-sweating. However, we suspect that most within-subject (across gland and test) variation in β/M reflects experimental variability. As noted, the largest variation across glands occurs because of gland size/secretory capacity [14], which respond to the environment [24, 25]. β-sweat rates are highly correlated to M-sweat rates, so that the average ratio, β/M, tended to be flat across glands within a subject (**Fig 2**), but still showed considerable within subject variation. Within a subject, as gland β/M ratios differed increasingly from the overall β/M mean their SD’s also tended to increase, consistent with experimental and random variability.

### Across-subject variation in β/M-sweat rates

Given that β/M varies within subjects, to what extent can across-subject variation (**Fig 3**) be attributed to intrinsic variation in CFTR activity? CFTR is rate-limiting for β-sweating, [6], and our hypothesis was that variation in β/M ratios across healthy control subjects would mainly reflect variations in CFTR activity arising from various polymorphisms in or around CFTR, many of which have been documented e.g. [26–31]. However, variation across glands within subjects was similar to variation across subjects, showing that a great deal of variation is due to factors other than genetic variation at the CFTR locus, in spite of efforts to minimize such factors. For example, to compensate for levels of β-receptors or other components of the cAMP pathway the stimulating cocktail was formulated at saturating concentrations to swamp any such differences [7]. This strategy couldn’t be used with the powerful cholinergic pathway, because the bubble assay dictates a low concentration of methacholine to avoid rapid merging of sweat bubbles. Thus, experimental variation in the M-sweat stimulus likely contributes to noise, but if individual differences in components of the cholinergic pathway were important factors we would have expected to see a trend toward lower β/M ratios with higher M sweat rates, but that was not observed (**Fig 3A**). In an analysis of sweat chloride measures among CF subjects, 44% of the variability was due to factors other than CFTR variation [5]. In this study of healthy subjects, we lack independent measures of variation in and around the CFTR locus, so our data are insufficient to apportion the observed variation between the CFTR-dependent and CFTR-independent factors. The best evidence for a substantial contribution from CFTR remains the 50% decrease seen in the mean β/M of carriers.

### Health consequences arising from individual differences in CFTR activity

In physiology and medicine generally, it is understood that when we express results as “percent of control” we are talking about an average control value. Control values for biological measures span ranges that can be wide. In **Fig 3B**, the ratio of β/M sweat responses were normalized to the average of all β-sweat/cholinergic sweat responses in the sample, giving a 7-fold range (**Table 2**). What is the significance of more accurately determining the variation of CFTR activity in healthy controls? Most CF phenotypic measures, such as sweat chloride levels or pulmonary function results, are not rate-limited by CFTR and show only modest variation in the control population. This is also true for measures like chloride based short-circuit current (I_sc_) across respiratory epithelia [32, 33]. However, unlike Cl^-^, HCO_3_^-^ conductance in the airways *is* rate-limited by CFTR activity [34]. That is important, because HCO_3_^-^ secretion increases and stabilizes the pH of airway surface liquid, which is necessary for optimal airway defenses against infections [35, 36]. Shah *et al*. proposed that the reduction of HCO_3_^-^ (but not Cl^-^) in airway secretions of CF carriers might account for why they have slightly but significantly elevated risk for various CF-related diseases [34].

As noted in the introduction, carriers have elevated risk for at least 57 conditions also seen in CF [2]. Our present and previous data on the distribution of CFTR activity among control subjects and carriers [6] and extrapolations from low CFTR transcripts in some healthy adults [27] indicate that the lower tail of the distribution of CFTR activity in some carriers might be < 10% of the control average. CF carriers in the lower tail of the distribution of CFTR activity may contribute most of the increased prevalence of CF-like disease among carriers shown by Miller *et al*. [2]. The prevalence of CF-like disease in CF carriers was very low, but was statistically greater than in the control group. For example, the prevalence of the following six conditions in carriers and controls respectively, was: bronchiectasis 0.288% vs. 0.052%; male infertility 7.139% vs. 1.558%; pancreatitis (acute) 0.874% vs. 0.353%; neonatal jaundice 4.494% vs. 2.470%; asthma 11.312% vs. 8.637%; and gastroesophageal reflux disease (GERD) 10.767% vs 9.450%, all significant at p < 0.001 [2]. The similar prevalence for some of these conditions in the *control* group might also result from lower levels of CFTR activity in unidentified carriers. Although the increased risk for carriers is slight, Miller *et al* [2]. point out that with more than 10 million CF carriers in the U.S. (and probably at least three times that number world-wide), even small risks can have large aggregate effects.

### Limitations

This study has important limitations. The healthy control sample was not genotyped, requiring an ad hoc estimate of carriers in the sample. We estimated a 78% chance of having ≤ 2 or carriers and a 98% chance of having ≤ 4 or fewer carriers. We estimated that having 4 carriers increased the mean by 3–6%, with modest effects on the parameters of the distribution. Other features of the cohort that could have been useful in retrospect were not recorded, such as age, body mass index, and ethnicity.

Sweat chloride was measured only once per subject, on only one arm, using in-lab (not clinical) sweat chloride measurements. A weak correlation between sweat chloride and the β/M ratio was expected, based on the logarithmic relation between sweat chloride and β-sweat measures which is quite flat among healthy controls [37]. A complete lack of a correlation was unexpected, but adds to the evidence that variation of both the β/M ratio and sweat chloride arises from multiple sources that obscure the contribution from CFTR variation. Random variation has been shown to be appreciable for sweat chloride measures within CF subjects [5] and even for CF subjects having the same CFTR mutations [31].

## Supporting information

S1 File Analysis of within-subject sweat rates (data for Fig 2). (XLSX)

S2 File Analysis of beta-sweating across subjects-i (data for Fig 3A). (XLSX)

S3 File Analysis of beta-sweating across subjects-ii (data for Fig 3B). (XLSX)

S4 File Analysis of sweat chloride values-i (data for Fig 4A). (XLSX)

S5 File Analysis of sweat chloride values-ii (data for Fig 4B). (XLSX)

S6 File Analysis of arm sizes and sweat rates (data for Fig 5A and 5B). (XLSX)

S7 File Analysis of sexes and sweat rates (data for Fig 5C and 5D). (XLSX)
